# A World’s Congress of Homœopathy

**Published:** 1893-01

**Authors:** Pemberton Dudley

**Affiliations:** General Secretary


### A WORLD'S CONGRESS OF HOMŒOPATHY.

*To the Members of the American Institute of Homœopathy.*

The General Secretary deems it proper to publish the following announcement:

At the recent session of the Institute it was announced that the United States Government had authorized the holding of a series of Congresses on subjects of a scientific and social character, during the continuance of the Columbian Exhibition in Chicago, in 1893. Among these there will be included a World's Congress of Homœopathic Physicians and Surgeons. The Art Building, now in course of erection, is for the free use of these Congresses, and for their Sectional Meetings, Committees, etc. The department of the exhibition known as the World's Congress Auxiliary has appointed a committee, consisting of a number of homœopathic physicians of Chicago, with Dr. J. S. Mitchell as its chairman, to prepare and arrange for the Congress of Homœopathy.

Acting on this announcement and on motion of Dr. Mitchell, the Institute appointed a committee to consider what action should be taken in reference to it. This committee afterward presented the following recommendations which the Institute adopted unanimously.

1. That the meetings of the Congress and of the Institute be held in conjunction after the plans of the previous Congresses.

2. That the officers elect of the Institute hold office for two years.

3. That the business meetings of the Institute be held daily during the continuance of the Congress and that it adjourn to meet with the Congress.

4. That the Sectional Meetings of the Institute Bureaus appointed at this session, and other scientific proceedings of the Institute, be deferred until the session of 1894.

The Institute has thus ordered that for next year its own sessions be limited to the transaction of its general business, and that all its scientific energies shall be devoted to the interest and success of the World's Congress.

At the Congress at Atlantic City in 1891 there was an attendance of 1,024 homœopathic physicians and visitors. At the Institute meeting in Washington in 1892 there were 881 members and visitors. There are good reasons to believe that the Chicago Congress will more than double the larger of these numbers. The indications of a large attendance from abroad are far more encouraging than in 1891.

During the past two years the Institute has added more than 400 names to its roll of membership notwithstanding the fact that the meetings were held within little more than a hundred miles of each other. The General Secretary considers it perfectly feasible to secure at least 400 more during the Chicago Congress and expects to labor earnestly and persistently to that end. He suggests that all societies, State and local, appoint committees to canvass their membership to secure larger representation in the National Society. This work should begin now. Blanks will be forwarded on application. College faculties should endeavor to secure members from among their alumni and thus enhance their collegiate influence in Institute Councils.

The Institute has adopted a resolution requesting investigations on the subject of Comparative Mortality Statistics in all our larger cities. One of our largest cities is already taking measures to this end through its county society.

All reports secured should be communicated to Dr. T. F. Smith, 264 Lenox Ave., New York City.

PEMBERTON DUDLEY, M. D.,
*General Secretary.*

